# Complementary therapies for the management of attention deficit hyperactivity disorder in children: a scoping review

**DOI:** 10.1590/1518-8345.7915.4701

**Published:** 2025-11-03

**Authors:** Lara Freire Szychta, Sara Emilly Lima Sombra, Glaubervania Alves Lima, Maria Williany Silva Ventura, Brena Shellem Bessa de Oliveira, Francisca Elisângela Teixeira Lima

**Affiliations:** 1 Universidade Federal do Ceará, Fortaleza, CE, Brazil.; 2 Scholarship holder at the Conselho Nacional de Desenvolvimento Científico e Tecnológico (CNPq), Brazil.; 3 Scholarship holder at the Coordenação de Aperfeiçoamento de Pessoal de Nível Superior (CAPES), Brazil.

**Keywords:** Attention Deficit Disorder with Hyperactivity, Complementary Therapies, Child, Infant, Child Health, Health Personnel

## Abstract

to map the scientific literature regarding complementary therapies used by health professionals for children with Attention Deficit Hyperactivity Disorder.

this scoping review was based on the recommendations proposed by the Joanna Briggs Institute. Five data sources were searched. Inclusion criteria comprised articles published in English, Spanish, or Portuguese; children aged one to nine years diagnosed with Attention Deficit Hyperactivity Disorder, regardless of additional age ranges covered; and no restrictions on publication date, study design, or bibliographic type. Therapies were grouped into four categories: mind-body therapies, supplementation, herbal therapy, and dietary therapy.

a total of 1,444 publications were identified, with 133 peer-reviewed articles selected for analysis. Sixty-five complementary therapies were identified, with neurofeedback (n=38) being the most frequently cited mind-body therapy. Other interventions included a prevalence of polyunsaturated fatty acid supplementation (n=14), *Ginkgo biloba* use (n=6) in phytotherapy, and oligo-antigenic diet (n=5) in dietary therapy.

complementary therapies show potential for alleviating symptoms of Attention Deficit Hyperactivity Disorder in childhood. However, some therapies still lack scientific validation, highlighting the need for targeted experimental studies to ensure safe and effective use.

## Highlights

**(1)** Several complementary therapies are used to control ADHD in children. **(2)** Complementary therapies can improve the core symptoms of ADHD. **(3)** More research should be conducted to ensure the efficacy of the therapies. **(4)** A professional approach to ADHD is individualized and holistic. **(5)** The health professional is responsible for health education and ADHD monitoring.

## Introduction

Attention-deficit/hyperactivity disorder (ADHD) is a neurodevelopmental disorder characterized by persistent symptoms of inattention, hyperactivity, and impulsivity, which impair childhood development^(1-2)^. It is estimated that TDAH affects approximately 3% to 8% of children in nearly all studied regions worldwide, and in more than half of cases, the condition persists into adulthood^(3-5)^. In the United States, the diagnosis rate among children reached 9.4%^(6)^. This disorder is generally more common in males than females, with an approximate ratio of 2:1 in children and 1.6:1 in adults. In addition, girls are more likely than boys to have predominantly inattentive characteristics^(7-8)^, such as difficulty maintaining attention, being easily distracted, and struggling to complete tasks^(9)^.

ADHD compromises personal, social, and emotional functioning during childhood, as well as affect family relationships due to challenges related to behavior, academic performance, and the constant need for specialized support^(10-12)^. ADHD treatment is multimodal and includes both pharmacological and psychological interventions^(13)^. First-line pharmacological treatment for ADHD includes the use of stimulants such as amphetamine (AMP) and methylphenidate (MPH), both of which have been shown to have comparable efficacy^(14)^. However, children treated with stimulants often experience negative side effects such as sleep disturbances, decreased appetite, headaches, irritability, and stomach pain^(15)^.

In addition, economic barriers within the healthcare system hinder adherence to pharmacological treatment. In Brazil, for instance, the absence of these medications from the National List of Essential Medicines (RENAME) limits their free distribution, restricting patient access to treatment^(16-17)^.

As a result, families and healthcare professionals have sought alternative approaches to complement the care of children with ADHD^(18-19)^. In this context, complementary therapies—defined by the National Center for Complementary and Integrative Health (NCCIH) in the United States as unconventional approaches used alongside conventional medicine to enhance treatment—have been increasingly discussed as supportive strategies for managing ADHD symptoms^(20-21)^.

These therapies include practices such as acupuncture, meditation, herbal medicine, and yoga, among others, and are incorporated into Brazil’s National Policy on Integrative and Complementary Practices (PNPIC), implemented within the Unified Health System (*Sistema Único de Saúde*, SUS) in 2006. Following this policy, the use of complementary therapies in Brazil increased by 70% in 2024 compared to 2022, particularly in primary healthcare and specialized services referred by professionals^(22-23)^.

Although these approaches still face challenges regarding acceptance, prescription, and effective implementation within healthcare services, studies have highlighted significant benefits, particularly in the treatment of children with ADHD. These benefits include a reduction in excessive movements^(24)^, improvements in selective and sustained attention^(25)^, enhanced concentration, motor skills^(26)^, and memory^(27-28)^.

In relation to healthcare professionals, obstacles such as the lack of specialized training in these practices, insufficient team support, absence of standardized clinical protocols, and institutional resistance to non-conventional approaches often limit the effective use of complementary therapies in managing children with ADHD—even when users express interest or evidence suggests potential benefits^(29-31)^.

Thus, it is essential to understand which complementary therapies are being promoted by healthcare teams as part of comprehensive care for children with ADHD. Given the growing interest in non-pharmacological approaches and the need to expand professional knowledge about complementary therapies, integrating multidimensional care into healthcare services becomes increasingly relevant. However, evidence on this topic remains scarce, underscoring the need for further research. Therefore, the aim of this study was to identify, in the scientific literature, the complementary therapies promoted by healthcare professionals for children with ADHD.

## Method

### Type of study

This is a scoping review conducted based on the methodological guidelines outlined by the Joanna Briggs Institute (JBI)^(32)^ and the PRISMA Extension for Scoping Reviews (PRISMA-ScR)^(33)^. The study protocol has been registered on the Open Science Framework (OSF) platform at osf.io/twdrh.

### Setting

The literature search was conducted in the following databases: Medical Literature Analysis and Retrieval System Online (MEDLINE/PubMed), Excerpta Medica Database (EMBASE), Scopus, Web of Science (WoS), and Latin American and Caribbean Health Sciences Literature (LILACS). In addition, a secondary search in Google Scholar and a manual reverse search of the references of the included primary studies were performed to expand the mapping.

### Study period

It was carried out between May 2023 and June 2023. Additionally, an updated literature search was conducted in April 2025 to ensure the review remains current.

### Population

The PCC mnemonic, representing population (P), concept (C), and context (C), was used to define the title and construct the review question, as recommended by the JBI for scoping reviews^(32)^. In this study, the population was defined as children aged one to nine years, according to the World Health Organization age classification^(34)^; the concept included complementary therapies administered by health professionals; and the context focused on the diagnosis of ADHD. Thus, the guiding question was formulated as follows: What complementary therapies are promoted by the health care team to control ADHD in children?

### Selection criteria

Inclusion criteria for studies were: addressing the guiding question; including children aged one to nine years, regardless of whether other age groups were also included; published in Portuguese, English or Spanish; without date restrictions; and of any methodological design and bibliographic material. Studies that covered the established age range and included other age groups were also considered.

**Figure 1-:**
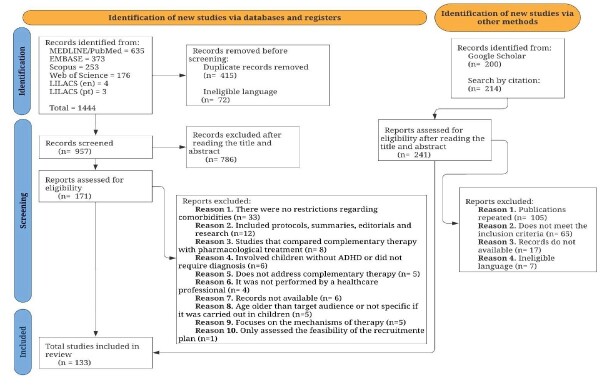
PRISMA flow diagram of study selection. Fortaleza, CE, Brazil, 2025

Exclusion criteria were: abstracts published in proceedings of scientific meetings; participants with suspected or diagnosed comorbid psychiatric disorders such as anxiety, depression, oppositional defiant disorder, autism spectrum disorder, among others; comparison of complementary therapy with pharmacological treatment; assessment of the feasibility of a recruitment plan; and focus solely on the technical mechanisms of therapy. These criteria are numbered from 1 to 10 in the PRISMA flowchart (Figure 1).

### Sample definition

The search Strategies included descriptors with proper truncation adapted to each database that described the population, complementary therapies, and ADHD diagnosis. Descriptors included: “child”, “children”, “complementary therapies”, “alternative medicine”, “alternative therapies”, “complementary medicine”, “attention deficit disorder with hyperactivity”, “ADHD”, “attention deficit disorder with hyperactivity”, “attention deficit hyperactivity disorder”, and alternative terms. See search strategy in Supplementary Appendix 1 (in https://doi.org/10.48331/scielodata.8ZOILU).

### Data collection

Search results were uploaded into the Rayyan software and duplicates were removed. Two independent reviewers screened titles and abstracts, followed by full-text analysis. Any disagreements were resolved by a third reviewer. This process resulted in a list of 108 studies to be included in the review. With the April 2025 update, 25 additional studies were added, resulting in a total of 133 studies included in this review.

### Study variables

The variables used were: author(s), year of publication, country, language, objective(s), type of study, population, complementary therapy, intervention duration and main results.

### Data processing and analysis

Descriptive statistics (frequencies and percentages) were used to summarize the relevant quantitative characteristics of the studies. Primary outcomes were grouped and presented in tables, graphs, and figures, and then discussed in accordance with the identified references.

### Ethical aspects

This study did not require approval from a Research Ethics Committee.

## Results

The database search identified 1,444 studies, of which 415 were duplicates and 72 were in ineligible languages, reducing the count to 957 articles. Of these, 786 studies were excluded for not meeting the inclusion criteria. The remaining 171 articles underwent a full-text review, resulting in the exclusion of 85 more, leaving a final sample of 86 studies for the review.

Additionally, grey literature searches using Google Scholar and citation tracking of the included studies identified 414 documents, from which 47 articles were incorporated into the final compilation. As a result, a total of 133 studies were included in this review, as illustrated in the PRISMA flowchart (Figure 1).

Regarding study characteristics, as shown in Table 1, the publication years ranged from 1979 to 2024, with 72.2% of the studies published between 2010 and 2024. Notably, there was a higher number of publications in the years 2024 (n=16), 2014 (n=14) and 2022 (n=10). Some studies were concentrated in Asia (37.6%), North America (30.9%), and Europe (26.3%). The search strategy used in this study did not identify productions in Central America. The review included a total of 8,798 children diagnosed with ADHD, with 51.1% (n=68) having 10 to 100 participants per study. The predominant language was English (97.8%). Among the methods used, 45 publications were randomized clinical trials, 39 were reviews—including 11 meta-analyses—11 were experimental studies, and 6 were systematic reviews. Notably, the studies classified as experimental did not specify their methodological design in etail. In 46.6% of the articles, the reported intervention period ranged from 1 to 6 months.

**Table 1-:** Characteristics of the included studies (n = 133). Fortaleza, CE, Brazil, 2025

**Characteristics**	**Number (%)**
Year	
< 2000	7 (5.2%)
2000-2009	30 (22.6%)
2010-2024	96 (72.2%)
Region	
Asia	50 (37.6%)
North America	41 (30.9%)
Europe	35 (26.3%)
Oceania	2 (1.5%)
South America	2 (1.5%)
Africa	3 (2.2%)
Language	
English	130 (97.8%)
Spanish	2 (1.5%)
Portuguese	1 (0.7%)
Study design	
Randomized clinical trial	45 (33.8%)
Review	39 (29.3%)
Experimental study	11 (8.3%)
Pilot study	6 (4.5%)
Qualitative study	4 (3%)
Case-control study	3 (2.3%)
Other	25 (18.8%)
Number of participants	
< 10	8 (6%)
10-100	68 (51.1%)
100-500	18 (13.6%)
> 500	4 (3%)
Not mentioned	35 (26.3%)
Duration of complementary therapies	
≤ 1 month	17 (12.8%)
1 to 6 months	62 (46.6%)
≥ 6 months	12 (9%)
Not specified	42 (31.6%)

**Figure 2-:**
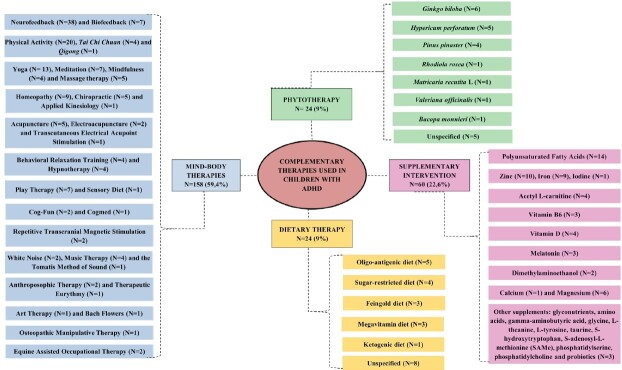
Complementary therapies found in studies and the number of articles for each therapy. Fortaleza, CE, Brazil, 2025

A total of 65 complementary therapies were identified, divided into four categories: mind-body therapies (n=158), supplementary intervention (n=60), phytotherapy (n=24), and dietary therapy (n=24), as shown in Figure 2. Among mind-body therapies, neurofeedback (n=38) was the most reported practice, followed by physical activity (n=20) and yoga (n=13). The most reported supplemental intervention was polyunsaturated fatty acid supplementation (n=14), followed by zinc (n=10) and iron (n=9). Other supplements included amino acids, glyconutrients, gamma-aminobutyric acid, glycine, L-theanine, L-tyrosine, taurine, 5-hydroxytryptophan, S-adenosyl-L-methionine (SAMe), phosphatidylserine, phosphatidylcholine and probiotics. Among the herbal therapies, *Ginkgo biloba* (n=6), *Hypericum perforatum* (n=5), and Pycnogenol (n=4) stood out. Among the dietary therapies, the oligo-antigenic diet (n=5) and the sugar-restricted diet (n=4) were emphasized.

A table with more characteristics and references of the included articles was included in Supplementary Material 2 (in https://doi.org/10.48331/scielodata.WMW6W7).

## Discussion

This scoping review identified 65 modalities of complementary therapies employed by healthcare professionals for children diagnosed with ADHD, categorized into four approaches: mind-body therapies, supplementary intervention, phytotherapy, and dietary therapy.

Among the mind-body therapies, biofeedback, neurofeedback, physical activity, and yoga stood out. Biofeedback is a therapeutic technique that regulates the autonomic nervous system’s response, leading to a balance of sympathetic and parasympathetic functions. This therapy aims to enable psychophysiological self-control in the patient^(35)^.

Neurofeedback is a subset of biofeedback that targets the Central Nervous System (CNS) and trains the patient to self-regulate their brain waves through stimulation and observation of brain activity^(36-37)^. Findings suggest the apparent efficacy of biofeedback for children with ADHD, resulting in improved attention^(38-40)^.

However, a recent meta-analysis did not confirm the effectiveness of neurofeedback as a treatment for ADHD^(41)^. In a new review, it is reported that meta-analyses have concluded that neurofeedback is effective in the short term for ADHD symptoms^(42)^.

In relation to physical activity, it is a practice that provides multiple benefits for both physical and mental health^(43)^. Aerobic exercise has been evaluated as a therapeutic intervention that can effectively alleviate symptoms of ADHD and promote improvements in memory and executive function^(27,44)^. Aerobic exercise, such as dancing, swimming, walking, and running, appears to reduce inattention, impulsivity, and hyperactivity in children with ADHD^(28)^.

A recent meta-analysis found that physical activity improves concentration and facilitates the development of motor skills^(45)^. Notably, physical activity has a more pronounced effect when compared to other non-pharmacological treatment modalities^(26)^. The findings reinforce physical activity as an effective complementary strategy for managing ADHD symptoms in pediatric populations.

Another therapy featured prominently in the literature is yoga, which is based on a series of exercises aimed at achieving physical and mental balance and represents a collection of techniques and knowledge aimed at personal fulfillment, psychological growth, and spiritual well-being^(46)^. An experimental study using pre- and post-test assessments to evaluate the role of yoga in children with ADHD reported positive effects, including reductions in movements such as hand and/or foot fidgeting and improvements in interruptive and intrusive behaviors during other activities^(47)^.

Perceptible improvements in selective and sustained attention, as well as discrimination skills, have been observed in children with ADHD following yoga practice^(24)^. As a result, recent studies indicate a positive attitude toward the use of yoga as a complementary therapy for ADHD.

Other complementary practices such as biofeedback, meditation, homeopathy, chiropractic care, acupuncture, massage therapy, *Tai Chi Chuan*, hypnotherapy, play therapy, mindfulness, behavioral relaxation training, cognitive function training, electroacupuncture, repetitive transcranial magnetic stimulation, white noise, anthroposophic therapy, art therapy, applied kinesiology, Cogmed, *Qigong*, therapeutic eurythmy, Bach flower remedies, the Tomatis method of sound training, music therapy, osteopathic manipulative therapy, equine-assisted therapy, sensory diet, and transcutaneous electrical acupoint stimulation have yielded inconclusive evidence in the pediatric population. This underscores the need for further research in this area to substantiate their efficacy.

One of the identified supplemental interventions is the use of polyunsaturated fatty acids, which consist of long-chain acids with multiple double bonds in their structural molecules, with omega-3 and omega-6 being the most prominent variants. The primary sources of these supplements include salmon, evening primrose oil, and vegetable oils such as corn oil^(25,48-49)^.

A randomized, double-blind, placebo-controlled trial conducted in Canada showed that omega-3 fatty acids showed statistically significant trends in reducing core symptoms of ADHD in children diagnosed with the disorder^(50)^. Similarly, another study observed incremental improvements in children’s academic performance^(51)^. However, a placebo-controlled trial in Italian children with ADHD found no significant difference in improved learning or reduced inattention with omega-3 and omega-6 supplementation compared to the placebo group^(52)^. Thus, it is clear that there is currently insufficient scientific evidence to support the use of omega-3 and omega-6 supplementation as a stand-alone therapy for ADHD.

Other components described in the literature include zinc and iron, minerals that are directly associated with physical and neurological development in individuals. These minerals serve various functions, with zinc being critical for cellular processes such as protein and DNA synthesis, and iron playing a role in the production of hormones such as dopamine and norepinephrine^(53)^.

Zinc supplementation has been shown to be effective in reducing hyperactivity and impulsivity in children^(54-56)^. However, its efficacy appears to be dose-dependent, which requires further scientific investigation^(57-58)^. A randomized clinical trial involving 60 Asian children showed that zinc supplementation did not result in significant differences between groups before and after the intervention, suggesting a lack of efficacy for this type of complementary therapy^(59)^.

Regarding iron supplementation, a randomized clinical trial found that children who received iron supplementation therapy showed progress in the treatment of ADHD, particularly in the subscales of hyperactivity, impulsivity, and inattention^(60)^. However, another review indicated that there is insufficient scientific evidence to recommend iron supplementation for children with ADHD who are not iron deficient^(61)^. Despite the diversity of literature on supplementation, confirmation of the use of these minerals as a therapy with a positive impact on the treatment of ADHD is still lacking.

Supplemental interventions have yielded mixed results with the use of magnesium, acetyl L-carnitine, vitamin B6, melatonin, vitamin D, dimethylaminoethanol, calcium and magnesium, iodine, glyconutrients, gamma-aminobutyric acid, glycine, L-theanine, L-tyrosine, taurine, 5-hydroxytryptophan, S-adenosyl-L-methionine (SAMe), phosphatidylserine, and phosphatidylcholine. Although some studies reported benefits in reducing ADHD symptoms, the evidence remains insufficient for routine clinical use, and additional controlled studies are necessary. Notably, no scientific support was found to justify the use of glyconutrients in treating ADHD.

*Ginkgo biloba* is an herbal remedy with antioxidant and anti-inflammatory properties that share similarities with the pharmacological class of nootropics, substances that act on the central nervous system to improve cognitive function^(62)^. It is used to treat various cognitive dysfunctions, including dementia, cerebral vascular insufficiency, recent memory loss, headaches, dizziness, and tinnitus^(63)^.

*Ginkgo biloba* has demonstrated an improved response to clinical treatment, although its effects were limited to symptoms of inattention in a six-week study^(64)^. However, despite its promising effects on ADHD symptoms, it yielded inconclusive results in another study^(65)^.

In the area of herbal therapy, other herbs such as *Hypericum perforatum*, *Pinus pinaster*, *Rhodiola rosea*, *Matricaria recutita* L., *Valeriana officinalis*, and *Bacopa monnieri* have been identified. There is currently insufficient scientific evidence to recommend these herbs as effective complementary therapies for ADHD and further research is needed.

An oligo-antigenic diet involves the consumption of foods without chemical additives, such as dyes and preservatives, that may trigger symptoms of ADHD by acting as potential antigens or food allergens. Commonly associated allergenic foods include cow’s milk, cheese, eggs, chocolate, and nuts^(66)^.

The evidence for restricting artificial food colorings has advanced to the point of being considered an evidence-based practice^(67)^. Results suggest that this diet, applied for 4 weeks, resulted in long-term improvements in ADHD symptoms, making it a valid complementary therapy for ADHD when reassessed approximately 3 years after therapy^(68)^. This therapy may serve as a useful adjunct to improve symptoms in pediatric ADHD.

Four other dietary practices have been mentioned, namely the sugar-restricted diet, the Feingold diet, the megavitamin diet, and the ketogenic diet. Other research studies reported conflicting and inconclusive results for the Feingold and ketogenic diets. Notably, studies did not recommend megavitamin therapy due to potential side effects associated with this dietary approach.

ADHD presents significant challenges related to motor, perceptual, cognitive, and behavioral alterations. Therefore, age-specific approaches must be adopted to ensure appropriate management and care^(69)^.

In early childhood, particularly around 12 months of age, changes in motor activity, emotional expressiveness, and language development become evident^(70)^. Between the ages of 3 and 7, the first indicative signs of ADHD emerge, although formal diagnosis often occurs later. During this period, typically hyperactive behavior is noticeable. Recommended management strategies for this stage include establishing consistent routines and implementing more structured teaching methods^(11)^.

Children are typically diagnosed around age 7 due to academic or behavioral difficulties. Comparative studies have shown that children with ADHD perform worse in social, emotional, and academic domains than their peers^(11)^.

Several limitations were encountered during the development of this study, including language restrictions during the source selection phase, studies with missing critical data, and unreadable articles. These limitations prevented a thorough examination of the data.

The role of nurses in primary healthcare clinics can positively influence the effectiveness of complementary therapies through health education targeted at both parents and children with ADHD. This educational process, combined with consultations that encourage active family participation in setting expectations, contributes to greater treatment adherence and strengthens the therapeutic bond with the patient^(71)^.

Furthermore, this role extends beyond primary health care, encompassing the school environment, where nurses monitor the child while considering behavioral, psychosocial, and educational aspects^(72-73)^.

It is expected that nurses, along with other health care professionals, will be able to incorporate these practices into their consultations as an adjunct to pharmacologic treatment. They can tailor these approaches to the individual needs of each patient, with the goal of providing comprehensive care and improving the overall quality of life for those undergoing treatment.

## Conclusion

The use of complementary therapies presents promising potential for improving symptoms of pediatric ADHD, with particular emphasis on mind-body therapies such as neurofeedback; in supplementation, the use of polyunsaturated fatty acids, zinc, and iron; in herbal therapy, the use of *Ginkgo biloba*; and in dietary therapy, the oligo-antigenic diet.

These interventions can help improve focus, behavioral regulation, motor skills, and academic performance in children diagnosed with ADHD, addressing aspects related to hyperactivity, impulsivity, and inattention.

However, due to the limited number of studies and the lack of recent research in certain therapies, the evidence regarding the effectiveness of some of these practices remains inconclusive.

The development of experimental studies, particularly for less commonly used therapies, may provide a theoretical basis for the safe use and recommendation of these therapies in clinical practice. In addition, such studies may lead to better outcomes in the treatment of ADHD in children.

## Data Availability

The dataset of this article is available on the RLAE page in the SciELO Data repository, at the links https://doi.org/10.48331/SCIELODATA.8ZOILU and https://doi.org/10.48331/SCIELODATA.WMW6W7.
