# Supplementary material for: Complementary therapies for the management of attention deficit hyperactivity disorder in children: a scoping review
Source: Rev Lat Am Enfermagem. 2025 Nov 3;33:e4701. doi: 10.1590/1518-8345.7915.4701 (PMC12587758; doi:10.1590/1518-8345.7915.4701)
Supplement: Translation

# Translation: Terapias complementares para o manejo do transtorno do déficit de atenção e hiperatividade em crianças: uma revisão de escopo

**DOI:** 10.1590/1518-8345.7915.4702

**Authors:** Lara Freire Szychta, Sara Emilly Lima Sombra, Glaubervania Alves Lima, Maria Williany Silva Ventura, Brena Shellem Bessa de Oliveira, Francisca Elisângela Teixeira Lima

**Affiliations:** 1 Universidade Federal do Ceará, Fortaleza, CE, Brasil.; 2 Bolsista do Conselho Nacional de Desenvolvimento Científico e Tecnológico (CNPq), Brasil.; 3 Bolsista da Coordenação de Aperfeiçoamento de Pessoal de Nível Superior (CAPES), Brasil.

**Keywords:** Transtorno do Deficit de Atenção com Hiperatividade, Terapias Complementares, Criança, Lactentes, Saúde da Criança, Profissional de Saúde

## Abstract

mapear a literatura científica referente as terapias complementares empregadas por profissionais de saúde para crianças com Transtorno do Déficit de Atenção e Hiperatividade.

esta revisão de escopo seguiu as recomendações propostas pelo *JBI*. Foram consultadas cinco bases de dados. Os critérios de inclusão abarcaram artigos publicados em inglês, espanhol e português; estudos com crianças de um a nove anos diagnosticadas com Transtorno do Déficit de Atenção e Hiperatividade, independentemente de incluírem outras faixas etárias; e ausência de restrições quanto à data de publicação, desenho do estudo ou tipo de documento. As terapias foram organizadas em quatro categorias: terapias mente-corpo, suplementação, fitoterapia e terapia dietética.

foram identificadas 1.444 publicações, das quais 133 artigos revisados por pares foram selecionados para análise. No total, foram identificadas 65 terapias complementares, sendo o *neurofeedback* (n=38) a terapia mente-corpo mais frequentemente citada. Outras intervenções incluíram a suplementação com ácidos graxos poli-insaturados (n=14), o uso de *Ginkgo biloba* (n=6) na fitoterapia e a dieta oligoantigênica (n=5) no contexto da terapia dietética.

as terapias complementares mostram potencial na redução dos sintomas do Transtorno do Déficit de Atenção e Hiperatividade na infância. No entanto, algumas dessas abordagens ainda carecem de validação científica, o que reforça a necessidade de estudos experimentais direcionados que garantam seu uso seguro e eficaz.

## Destaques

**(1)** Diversas terapias complementares são utilizadas no controle do TDAH em crianças.**(2)** Essas terapias podem contribuir para a melhora dos sintomas centrais do transtorno. **(3)** É necessário realizar mais pesquisas para comprovar a eficácia dessas abordagens. **(4)** O cuidado profissional voltado ao TDAH deve ser individualizado e integral. **(5)** O profissional de saúde é responsável pela educação em saúde e pelo acompanhamento do TDAH.

## Introdução

O Transtorno do Déficit de Atenção e Hiperatividade (TDAH) é um transtorno do neurodesenvolvimento caracterizado por sintomas persistentes de desatenção, hiperatividade e impulsividade, que comprometem o desenvolvimento infantil^(1-2)^. Estima-se que o TDAH afete entre 3% e 8% das crianças nas diversas regiões analisadas mundialmente, sendo que, em mais da metade dos casos, o transtorno persiste na vida adulta^(3-5)^. Nos Estados Unidos da América (EUA) a taxa de diagnóstico entre crianças chegou a 9,4%^(6)^. De modo geral, o transtorno é mais prevalente no sexo masculino, com uma proporção aproximada de 2:1 na infância e 1,6:1 na vida adulta. Além disso, meninas tendem a apresentar mais frequentemente o subtipo predominantemente desatento^(7-8)^, manifestando dificuldade para manter o foco, tendência à distração e dificuldade para concluir tarefas^(9)^.

O TDAH compromete o funcionamento pessoal, social e emocional na infância, além de impactar negativamente as relações familiares, em virtude dos desafios comportamentais, do desempenho acadêmico e da constante necessidade de apoio especializado^(10-12)^. O tratamento do TDAH é multimodal, incluindo intervenções farmacológicas e psicológicas^(13)^. O tratamento medicamentoso de primeira linha envolve o uso de estimulantes como a anfetamina (AMP) e o metilfenidato (MPH), ambos com eficácia semelhante^(14)^. No entanto, o uso desses medicamentos em crianças pode ocasionar efeitos adversos, como distúrbios do sono, redução do apetite, cefaleia, irritabilidade e dor abdominal^(15)^.

Ademais, barreiras econômicas dentro do sistema de saúde dificultam a adesão ao tratamento farmacológico. No Brasil, por exemplo, a ausência desses medicamentos na Relação Nacional de Medicamentos Essenciais (RENAME) impede sua distribuição gratuita, limitando o acesso dos pacientes ao tratamento^(16-17)^.

Diante desse cenário, famílias e profissionais de saúde têm buscado abordagens alternativas que complementem o cuidado prestado a crianças com TDAH^(18-19)^. Nesse contexto, as terapias complementares — definidas pelo *National Center for Complementary and Integrative Health* (NCCIH), dos EUA, como abordagens não convencionais utilizadas em conjunto com a medicina tradicional para potencializar o tratamento — vêm sendo discutidas como estratégias auxiliares na gestão dos sintomas do TDAH^(20-21)^.

Essas terapias incluem práticas como acupuntura, meditação, fitoterapia e ioga, entre outras, e estão previstas na Política Nacional de Práticas Integrativas e Complementares (PNPIC), implementada no âmbito do Sistema Único de Saúde (SUS) em 2006. A partir dessa política, o uso de terapias complementares no Brasil cresceu 70% em 2024, em comparação com 2022, especialmente na atenção primária e em serviços especializados mediante encaminhamento profissional^(22-23)^.

Embora essas abordagens ainda enfrentem desafios relacionados à aceitação, à prescrição e à efetivação nos serviços de saúde, estudos têm apontado benefícios significativos, sobretudo no tratamento de crianças com TDAH. Entre os principais resultados observam-se a redução de movimentos excessivos^(24)^, a melhora da atenção seletiva e sustentada^(25)^, o aumento da concentração, o desenvolvimento de habilidades motoras^(26)^ e o aprimoramento da memória^(27-28)^.

Com relação aos profissionais de saúde, obstáculos como a ausência de capacitação específica nessas práticas, a falta de apoio das equipes, a falta de protocolos clínicos padronizados e a resistência institucional a abordagens não convencionais dificultam a aplicação efetiva das terapias complementares no manejo de crianças com TDAH, mesmo diante do interesse dos usuários ou da existência de evidências favoráveis^(29-31)^.

Torna-se, portanto, fundamental compreender quais terapias complementares têm sido promovidas pelas equipes de saúde como parte do cuidado integral a crianças com TDAH. Diante do crescente interesse por abordagens não farmacológicas e da necessidade de ampliar o conhecimento profissional sobre essas práticas, a integração de um cuidado multidimensional aos serviços de saúde mostra-se cada vez mais relevante. Entretanto, as evidências sobre o tema ainda são limitadas, o que ressalta a importância de novos estudos. Assim, este estudo teve como objetivo mapear na literatura científica as terapias complementares empregadas por profissionais de saúde para crianças com TDAH.

## Método

### Tipo de estudo

Trata-se de uma revisão de escopo realizada com base nas diretrizes recomendadas pelo *Joanna Briggs Institute* (JBI)^(32)^ e pela PRISMA *Extension for Scoping Reviews* (PRISMA-ScR)^(33)^. O protocolo do estudo foi registrado na plataforma *Open Science Framework* (OSF), disponível em osf.io/twdrh.

### Localização

A busca bibliográfica foi realizada nas seguintes fontes de informações: *Medical Literature Analysis* and *Retrieval System Online* (MEDLINE/PubMed), *Excerpta Medica Database* (EMBASE), *Scopus*, *Web of Science* (WoS) e Literatura Latino-Americana e do Caribe em Ciências da Saúde (LILACS). Além disso, foi feita uma busca secundária no *Google Scholar* e uma pesquisa manual reversa nas referências dos estudos primários incluídos, com o objetivo de ampliar o mapeamento.

### Período do estudo

A pesquisa foi conduzida entre maio e junho de 2023. Uma atualização da busca foi realizada em abril de 2025, a fim de garantir a atualidade da revisão.

### População

Foi utilizado o mnemônico PCC, que representa população (P), conceito (C) e contexto (C), para definir o título e estruturar a pergunta da revisão, conforme recomendação do JBI para revisões de escopo^(32)^. Neste estudo, a população foi composta por crianças com idade entre um e nove anos, de acordo com a classificação etária da Organização Mundial da Saúde^(34)^; o conceito abrangeu as terapias complementares realizadas por profissionais de saúde; e o contexto foi centrado no diagnóstico de TDAH. A partir disso, a pergunta norteadora foi formulada da seguinte forma: quais terapias complementares são empregadas pela equipe de saúde para o controle do TDAH em crianças?

### Critérios de seleção

Foram incluídos estudos que respondessem à pergunta norteadora; que envolvessem crianças com idade entre um e nove anos, ainda que outras faixas etárias também fossem contempladas; publicados em português, inglês e espanhol; sem restrições quanto ao ano de publicação; e de qualquer delineamento metodológico ou tipo de material bibliográfico. Estudos que abordassem a faixa etária estabelecida em conjunto com outras idades também foram considerados.

Os critérios de exclusão foram: resumos publicados em anais de eventos científicos; participantes com suspeita ou diagnóstico de transtornos psiquiátricos comórbidos, como ansiedade, depressão, transtorno opositor desafiante, transtorno do espectro autista, entre outros; comparação entre terapia complementar e tratamento farmacológico; avaliação da viabilidade de plano de recrutamento; e foco exclusivo nos mecanismos técnicos da terapia. Esses critérios estão numerados de 1 a 10 no fluxograma PRISMA (Figura 1).

### Definição da amostra

As estratégias de busca incluíram descritores com truncamentos apropriados, adaptados a cada base de dados, relacionados à população, às terapias complementares e ao diagnóstico de TDAH. Entre os descritores utilizados, destacam-se: “child”, “children”, “complementary therapies”, “alternative medicine”, “alternative therapies”, “complementary medicine”, “attention deficit disorder with hyperactivity”, “ADHD”, “attention deficit hyperactivity disorder”, entre outros termos alternativos. A estratégia completa está disponível no Apêndice Suplementar 1 (em https://doi.org/10.48331/scielodata.8ZOILU).

### Coleta de dados

Os resultados das buscas foram inseridos no *software Rayyan*, sendo posteriormente eliminadas as duplicatas. A triagem dos títulos e resumos foi realizada por dois revisores de forma independente, seguida da leitura dos textos completos. Divergências foram resolvidas por um terceiro revisor. Ao final desse processo, 108 estudos foram incluídos na revisão. Com a atualização realizada em abril de 2025, foram adicionados mais 25 estudos, totalizando 133 publicações incluídas na análise.

### Variáveis do estudo

As variáveis analisadas foram: autor(es), ano de publicação, país, idioma, objetivo(s), tipo de estudo, população, terapia complementar, duração da intervenção e principais resultados.

### Processamento e análise de dados

Para a descrição das características quantitativas dos estudos, foram utilizadas estatísticas descritivas (frequências e percentuais). Os desfechos primários foram agrupados e apresentados em tabelas, gráficos e figuras, sendo posteriormente discutidos à luz das referências identificadas.

### Aspectos éticos

Este estudo não exigiu aprovação por Comitê de Ética em Pesquisa.

## Resultados

Na busca nas fontes de informações foram identificados 1.444 estudos, dos quais 415 eram duplicados e 72 estavam em idiomas não elegíveis, reduzindo o total para 957 artigos. Desses, 786 estudos foram excluídos por não atenderem aos critérios de inclusão. Os 171 artigos restantes passaram por leitura na íntegra, o que resultou na exclusão de mais 85, restando uma amostra final de 86 estudos para a revisão.

Adicionalmente, buscas na literatura cinzenta utilizando o *Google Scholar* e o rastreamento de citações dos estudos incluídos identificaram 414 documentos, dos quais 47 foram incorporados à amostra final. Assim, esta revisão incluiu um total de 133 estudos, conforme ilustrado no fluxograma PRISMA (Figura 1).

**Figura 1-:** Fluxograma da busca na literatura e dos critérios de seleção dos estudos adaptado do PRISMA. Fortaleza, CE, Brasil, 2025

Em relação às características dos estudos, conforme Tabela 1, os anos de publicação variaram de 1979 a 2024, sendo que 72,2% dos estudos foram publicados entre 2010 e 2024. Observou-se maior número de publicações nos anos de 2024 (n=16), 2014 (n=14) e 2022 (n=10). Os estudos estavam concentrados, majoritariamente, na Ásia (37,6%), América do Norte (30,9%) e Europa (26,3%). A estratégia de busca utilizada nesta revisão não identificou publicações provenientes da América Central. A revisão incluiu um total de 8.798 crianças diagnosticadas com TDAH, sendo que 51,1% dos estudos (n=68) apresentaram entre 10 e 100 participantes. O idioma predominante das publicações foi o inglês (97,8%). Quanto ao delineamento metodológico, 45 publicações eram ensaios clínicos randomizados, 39 eram revisões (incluindo 11 metanálises), 11 eram estudos experimentais e 6 eram revisões sistemáticas. Vale destacar que os estudos classificados como experimentais não detalharam o desenho metodológico utilizado. Em 46,6% dos artigos, o tempo de intervenção relatado variou de um a seis meses.

**Tabela 1-:** Características dos estudos incluídos (n = 133). Fortaleza, CE, Brasil, 2025

**Características**	**Número (%)**
Ano	
< 2000	7 (5,2%)
2000-2009	30 (22,6%)
2010-2024	96 (72,2%)
Região	
Ásia	50 (37,6%)
América do Norte	41 (30,9%)
Europa	35 (26,3%)
Oceania	2 (1,5%)
América do Sul	2 (1,5%)
África	3 (2,2%)
Idioma	
Inglês	130 (97,8%)
Espanhol	2 (1,5%)
Português	1 (0,7%)
Delineamento do estudo	
Ensaio clínico randomizado	45 (33,8%)
Revisão	39 (29,3%)
Estudo experimental	11 (8,3%)
Estudo-piloto	6 (4,5%)
Estudo qualitativo	4 (3%)
Estudo caso-controle	3 (2,3%)
Outros	25 (18,8%)
Número de participantes	
< 10	8 (6%)
10-100	68 (51,1%)
100-500	18 (13,6%)
> 500	4 (3%)
Não informado	35 (26,3%)
Duração das terapias complementares	
≤ 1 mês	17 (12,8%)
1 a 6 meses	62 (46,6%)
≥ 6 meses	12 (9%)
Não especificado	42 (31,6%)

Foram identificadas 65 terapias complementares, agrupadas em quatro categorias: terapias mente-corpo (n=158), intervenções suplementares (n=60), fitoterapia (n=24) e terapia alimentar (n=24), conforme Figura 2. Dentre as terapias mente-corpo, a mais relatada foi o *neurofeedback* (n=38), seguida por atividade física (n=20) e yoga (n=13). A intervenção suplementar mais mencionada foi a suplementação com ácidos graxos poli-insaturados (n=14), seguida por zinco (n=10) e ferro (n=9). Outros suplementos incluíram aminoácidos, gliconutrientes, ácido gama-aminobutírico, glicina, L-teanina, L-tirosina, taurina, 5-hidroxitriptofano, S-adenosil-L-metionina (SAMe), fosfatidilserina, fosfatidilcolina e probióticos. Entre as terapias fitoterápicas, destacaram-se o *Ginkgo biloba* (n=6), o *Hypericum perforatum* (n=5) e o Pycnogenol (n=4). Em relação às terapias alimentares, ressaltaram-se a dieta oligoantigênica (n=5) e a dieta com restrição de açúcar (n=4).

Uma tabela contendo as características dos artigos incluidos na revisão e suas referências está disponível como Apêndice Suplementar 2 (em https://doi.org/10.48331/scielodata.WMW6W7).

**Figura 2-:** Terapias complementares identificadas nos estudos e número de artigos por tipo de terapia. Fortaleza, CE, Brasil, 2025

## Discussão

Esta revisão de escopo identificou 65 modalidades de terapias complementares empregadas por profissionais da saúde no tratamento de crianças com diagnóstico de TDAH, categorizadas em quatro abordagens: terapias mente-corpo, intervenções suplementares, fitoterapia e terapia dietética.

Entre as terapias mente-corpo, destacaram-se o *biofeedback*, o *neurofeedback*, a atividade física e a yoga. O *biofeedback* é uma técnica terapêutica que regula a resposta do sistema nervoso autônomo, promovendo o equilíbrio entre as funções simpáticas e parassimpáticas. Essa terapia busca capacitar o paciente ao autocontrole psicofisiológico^(35)^.

O *neurofeedback* é uma subcategoria da técnica terapêutica do *biofeedback* que atua no sistema nervoso central, treinando o paciente a autorregular suas ondas cerebrais por meio da estimulação e observação da atividade cerebral^(36-37)^. Os achados sugerem uma eficácia aparente do *biofeedback* em crianças com TDAH, resultando em melhora da atenção^(38-40)^.

No entanto, uma metanálise recente não confirmou a efetividade do *neurofeedback* como tratamento para o TDAH^(41)^. Em nova revisão, relatou-se que metanálises concluíram que o *neurofeedback* apresenta eficácia no curto prazo para sintomas do TDAH^(42)^.

Reconhece-se que atividade física é uma prática que proporciona benefícios múltiplos à saúde física e mental^(43)^. O exercício aeróbico tem sido avaliado como intervenção terapêutica capaz de aliviar sintomas do TDAH e promover melhorias na memória e nas funções executivas^(27,44)^. Exercícios aeróbicos, como dança, natação, caminhada e corrida, parecem reduzir desatenção, impulsividade e hiperatividade em crianças com TDAH^(28)^.

Uma metanálise recente mostrou que a atividade física melhora a concentração e favorece o desenvolvimento de habilidades motoras^(45)^. Notavelmente, a atividade física apresenta efeito mais expressivo quando comparada a outras modalidades de tratamento não farmacológico^(26)^. Os achados reforçam a atividade física como estratégia complementar eficaz para o manejo dos sintomas do TDAH em populações pediátricas.

Outra terapia amplamente abordada na literatura é a yoga, que se baseia em uma série de exercícios voltados ao equilíbrio físico e mental, representando um conjunto de técnicas e saberes voltados ao desenvolvimento pessoal, crescimento psicológico e bem-estar espiritual^(46)^. Um estudo experimental com avaliação pré e pós-intervenção sobre a prática de yoga em crianças com TDAH relatou efeitos positivos, incluindo redução de movimentos como balançar mãos e/ou pés e melhora de comportamentos interruptivos e intrusivos durante outras atividades^(47)^.

Melhoras perceptíveis na atenção seletiva e sustentada, assim como nas habilidades de discriminação, foram observadas após a prática de yoga em crianças com TDAH^(24)^. Assim, estudos recentes indicam uma atitude positiva frente ao uso da yoga como terapia complementar para o TDAH.

Outras práticas complementares como meditação, homeopatia, quiropraxia, acupuntura, massoterapia, *Tai Chi Chuan*, hipnoterapia, ludoterapia, *mindfulness*, treinamento em relaxamento comportamental, treinamento de funções cognitivas, eletroacupuntura, estimulação magnética transcraniana repetitiva, ruído branco, terapia antroposófica, arteterapia, cinesiologia aplicada, Cogmed, *Qigong*, euritmia terapêutica, florais de Bach, método Tomatis de treinamento sonoro, musicoterapia, terapia manipulativa osteopática, equoterapia, dieta sensorial e estimulação elétrica transcutânea de pontos de acupuntura apresentaram evidências inconclusivas na população pediátrica. Isso ressalta a necessidade de mais pesquisas para comprovar sua eficácia.

Em relação a intervenções suplementares identificadas, destaca-se o uso de ácidos graxos poli-insaturados, compostos por cadeias longas com múltiplas ligações duplas, sendo os principais representantes os ômega-3 e ômega-6. As principais fontes desses suplementos incluem salmão, óleo de prímula e óleos vegetais como o óleo de milho^(25,48-49)^.

Um ensaio clínico randomizado, duplo-cego e controlado por placebo realizado no Canadá demonstrou que os ácidos graxos ômega-3 apresentaram tendência estatisticamente significativa na redução dos sintomas centrais do TDAH em crianças diagnosticadas com o transtorno^(50)^. De forma semelhante, outro estudo observou melhorias graduais no desempenho escolar das crianças^(51)^. Entretanto, um ensaio clínico placebo-controlado com crianças italianas diagnosticadas com TDAH não encontrou diferença significativa na aprendizagem ou na redução da desatenção entre os grupos que receberam suplementação com ômega-3 e ômega-6 em comparação ao placebo^(52)^. Assim, verifica-se que não há evidência científica suficiente para sustentar o uso dos ômega-3 e ômega-6 como terapia isolada para o TDAH.

Outros componentes descritos na literatura incluem o zinco e o ferro, minerais diretamente associados ao desenvolvimento físico e neurológico. Esses minerais exercem diversas funções, sendo o zinco essencial para processos celulares como a síntese de proteínas e DNA, e o ferro fundamental para a produção de hormônios como a dopamina e a norepinefrina^(53)^.

A suplementação com zinco contribui para a redução da hiperatividade e impulsividade em crianças^(54-56)^. No entanto, sua eficácia parece estar relacionada à dosagem, exigindo maior investigação científica^(57-58)^. Um ensaio clínico randomizado com 60 crianças asiáticas mostrou que a suplementação com zinco não resultou em diferenças significativas entre os grupos antes e depois da intervenção, sugerindo ainda que a sua eficácia precisa de comprovação cientifica^(59)^.

Em relação à suplementação com ferro, um ensaio clínico randomizado mostrou que crianças que receberam essa intervenção apresentaram avanços no tratamento do TDAH, especialmente nas subescalas de hiperatividade, impulsividade e desatenção^(60)^. Contudo, outra revisão indicou que não há evidência científica suficiente para recomendar a suplementação com ferro em crianças com TDAH que não apresentam deficiência desse mineral^(61)^. Apesar da diversidade da literatura sobre suplementação, ainda falta comprovação da eficácia desses minerais como terapias com impacto positivo no tratamento do TDAH.

Intervenções suplementares apresentaram resultados variados com o uso de magnésio, acetil L-carnitina, vitamina B6, melatonina, vitamina D, dimetilaminoetanol, cálcio e magnésio, iodo, gliconutrientes, ácido gama-aminobutírico, glicina, L-teanina, L-tirosina, taurina, 5-hidroxitriptofano, S-adenosil-L-metionina (SAMe), fosfatidilserina e fosfatidilcolina. Embora alguns estudos tenham relatado benefícios na redução dos sintomas do TDAH, as evidências ainda são insuficientes para sua utilização rotineira na prática clínica, sendo necessários mais estudos controlados. Destaca-se que não foi encontrado suporte científico para justificar o uso de gliconutrientes no tratamento do TDAH.

Quanto aos fitoterápicos, o *Ginkgo biloba* possui propriedades antioxidantes e anti-inflamatórias, semelhante à classe farmacológica dos nootrópicos, substâncias que atuam no sistema nervoso central para aprimorar as funções cognitivas^(62)^. É utilizado no tratamento de disfunções cognitivas diversas, como demência, insuficiência vascular cerebral, perda recente de memória, cefaleia, tontura e zumbido^(63)^.

A utilização do *Ginkgo biloba* mostrou melhora na resposta ao tratamento clínico, embora seus efeitos tenham se limitado a sintomas de desatenção em um estudo de seis semanas^(64)^. No entanto, apesar dos efeitos promissores sobre os sintomas do TDAH, os resultados foram inconclusivos em outro estudo^(65)^.

Na área da fitoterapia, outras plantas como *Hypericum perforatum*, *Pinus pinaster*, *Rhodiola rosea*, *Matricaria recutita L.*, *Valeriana officinalis* e *Bacopa monnieri* também foram identificadas. Atualmente, não há evidência científica suficiente para recomendar essas plantas como terapias complementares eficazes para o TDAH, sendo necessárias novas pesquisas.

No que se refere à terapia dietética, a dieta oligoantigênica consiste no consumo de alimentos sem aditivos químicos, como corantes e conservantes, que podem desencadear sintomas do TDAH ao atuarem como potenciais antígenos ou alérgenos alimentares. Alimentos frequentemente associados a reações alérgicas incluem leite de vaca, queijo, ovos, chocolate e oleaginosas^(66)^.

As evidências relacionadas à restrição de corantes alimentares artificiais avançaram a ponto de serem consideradas uma prática baseada em evidências^(67)^. Os resultados sugerem que essa dieta, aplicada por quatro semanas, gerou melhorias duradouras nos sintomas do TDAH, sendo considerada uma terapia complementar válida quando reavaliada aproximadamente três anos após a intervenção^(68)^. Essa terapia pode servir como recurso auxiliar na melhora dos sintomas em crianças com TDAH.

Outras quatro práticas dietéticas foram mencionadas: a dieta com restrição de açúcar, a dieta Feingold, a dieta com megavitaminas e a dieta cetogênica. Estudos relataram resultados conflitantes e inconclusivos para as dietas Feingold e cetogênica. Notavelmente, os estudos não recomendaram a terapia com megavitaminas devido aos potenciais efeitos adversos associados a essa abordagem dietética.

Assim, o TDAH impõe desafios significativos relacionados a alterações motoras, perceptivas, cognitivas e comportamentais. Por isso, abordagens específicas para cada faixa etária devem ser adotadas para garantir um manejo e cuidado apropriados^(69)^.

Na primeira infância, especialmente por volta dos 12 meses de idade, tornam-se evidentes mudanças na atividade motora, na expressividade emocional e no desenvolvimento da linguagem^(70)^. Entre três e sete anos, surgem os primeiros sinais indicativos do TDAH, embora o diagnóstico formal ocorra posteriormente. Nessa fase, é comum a manifestação de comportamentos hiperativos. As estratégias recomendadas para esse estágio incluem o estabelecimento de rotinas consistentes e a adoção de métodos de ensino mais estruturados^(11)^.

O diagnóstico do TDAH ocorre, geralmente, por volta dos sete anos, em função de dificuldades escolares ou comportamentais. Estudos comparativos demonstraram que crianças com TDAH apresentam desempenho inferior aos seus pares nos domínios social, emocional e acadêmico^(11)^.

Durante o desenvolvimento deste estudo, diversas limitações foram identificadas, como a restrição linguística na seleção das fontes, ausência de dados relevantes em alguns estudos e artigos ilegíveis. Tais limitações impediram uma análise mais aprofundada das evidências.

O papel da enfermagem nas unidades de atenção primária à saúde pode interferir positivamente na efetividade das terapias complementares por meio da educação em saúde direcionada a pais e crianças com TDAH. Esse processo educativo, aliado a consultas que incentivam a participação ativa da família na definição de expectativas, contribui para uma maior adesão ao tratamento e fortalecimento do vínculo terapêutico com o paciente^(71)^.

Além disso, esse papel estende-se ao ambiente escolar, onde os profissionais de enfermagem acompanham a criança, considerando aspectos comportamentais, psicossociais e pedagógicos^(72-73)^.

Espera-se que enfermeiros, em conjunto com os demais profissionais da saúde, sejam capazes de incorporar essas práticas em suas consultas como recurso complementar ao tratamento farmacológico. Essas abordagens podem ser adaptadas às necessidades individuais de cada paciente, com o objetivo de proporcionar um cuidado integral e melhorar a qualidade de vida ao longo do tratamento.

## Conclusão

O uso de terapias complementares apresenta potencial promissor para a melhora dos sintomas do TDAH infantil, com destaque especial para as terapias mente-corpo, como o *neurofeedback*; no campo da suplementação, o uso de ácidos graxos poli-insaturados, zinco e ferro; na fitoterapia, a utilização de *Ginkgo biloba*; e, entre as terapias dietéticas, a dieta oligoantigênica.

Essas intervenções podem contribuir para a melhora da atenção, da regulação comportamental, das habilidades motoras e do desempenho acadêmico em crianças diagnosticadas com TDAH, abordando aspectos relacionados à hiperatividade, impulsividade e desatenção. Entretanto, devido ao número limitado de estudos e à ausência de pesquisas recentes sobre determinadas terapias, as evidências quanto à eficácia de algumas dessas práticas ainda são inconclusivas.

O desenvolvimento de estudos experimentais, especialmente voltados para terapias menos utilizadas, pode oferecer base teórica para o uso seguro e a recomendação dessas práticas na atuação clínica. Além disso, tais estudos podem contribuir para melhores desfechos no tratamento do TDAH infantil.

## Data Availability

O conjunto de dados deste artigo está disponível na página da RLAE no repositório SciELO Data, nos links https://doi.org/10.48331/SCIELODATA.8ZOILU e https://doi.org/10.48331/SCIELODATA.WMW6W7.

# Translation: Terapias complementarias para el manejo del trastorno por déficit de atención e hiperactividad en niños: revisión de alcance

**DOI:** 10.1590/1518-8345.7915.4700

**Authors:** Lara Freire Szychta, Sara Emilly Lima Sombra, Glaubervania Alves Lima, Maria Williany Silva Ventura, Brena Shellem Bessa de Oliveira, Francisca Elisângela Teixeira Lima

**Affiliations:** 1 Universidade Federal do Ceará, Fortaleza, CE, Brasil.; 2 Becaria del Conselho Nacional de Desenvolvimento Científico e Tecnológico (CNPq), Brasil.; 3 Becaria de la Coordenação de Aperfeiçoamento de Pessoal de Nível Superior (CAPES), Brasil.

**Keywords:** Trastorno por Déficit de Atención con Hiperactividad, Terapias Complementarias, Niño, Lactante, Salud Infantil, Personal de Salud

## Abstract

mapear la literatura científica sobre terapias complementarias promovidas por profesionales de la salud para niños con Trastorno por Déficit de Atención e Hiperactividad.

esta revisión de alcance siguió las recomendaciones propuestas por el *Joanna Briggs Institute*. Se consultaron cinco bases de datos. Los criterios de inclusión incluyeron artículos publicados en inglés, español o portugués; estudios con niños de uno a nueve años de edad diagnosticados con Trastorno por Déficit de Atención e Hiperactividad, más allá de que incluyan otros grupos de edad; y ausencia de restricciones en cuanto a fecha de publicación, diseño del estudio o tipo de documento. Las terapias se organizaron en cuatro categorías: terapias mente-cuerpo, suplementación, fitoterapia y terapia dietética.

se identificaron 1.444 publicaciones, de las cuales 133 artículos revisados por pares fueron seleccionados para su análisis. En total, se identificaron 65 terapias complementarias, siendo el *neurofeedback* (n=38) la terapia mente-cuerpo citada con mayor frecuencia. Otras intervenciones incluyeron la suplementación con ácidos grasos poliinsaturados (n=14), el uso de *Ginkgo biloba* (n=6) en fitoterapia y la dieta oligoantigénica (n=5) en el contexto de la terapia dietética.

las terapias complementarias demuestran potencial para reducir los síntomas del Trastorno por Déficit de Atención e Hiperactividad en la infancia. Sin embargo, algunos de estos enfoques aún requieren validación científica, lo que refuerza la necesidad de realizar estudios experimentales específicos para garantizar su uso seguro y eficaz.

## Destacados

**(1)** Se utilizan varias terapias complementarias para controlar el TDAH en niños. **(2)** Estas terapias pueden contribuir a mejorar los síntomas centrales del trastorno. **(3)** Son necesarias más investigaciones para demostrar la eficacia de estos enfoques. **(4)** La atención profesional centrada en el TDAH debe ser individualizada e integral. **(5)** El profesional sanitario es responsable de la educación sanitaria y del seguimiento del TDAH.

## Introducción

El trastorno por déficit de atención e hiperactividad (TDAH) es un trastorno del neurodesarrollo caracterizado por síntomas persistentes de inatención, hiperactividad e impulsividad, que comprometen el desarrollo infantil^(1-2)^. Se estima que el TDAH afecta entre un 3% y un 8% de los niños en las distintas regiones analizadas a nivel mundial, advirtiéndose que en más de la mitad de los casos el trastorno persiste hasta la edad adulta^(3-5)^. En Estados Unidos, la tasa de diagnóstico entre niños alcanzó el 9,4 %^(6)^. En general, el trastorno es más frecuente en personas del sexo masculino, con una proporción aproximada de 2:1 en la infancia y 1,6:1 en la edad adulta. Además, las niñas tienden a presentar con mayor frecuencia el subtipo predominantemente inatento^(7-8)^, mostrando dificultad para mantener la atención, tendencia a la distracción y dificultad para completar tareas^(9)^.

El TDAH compromete el funcionamiento personal, social y emocional en la infancia, además de impactar negativamente las relaciones familiares, debido a factores como trastornos de conducta, rendimiento académico y necesidad constante de apoyo especializado^(10-12)^. El tratamiento del TDAH es multimodal e incluye intervenciones farmacológicas y psicológicas^(13)^. El tratamiento farmacológico de primera línea implica el uso de estimulantes como la anfetamina (AMP) y el metilfenidato (MPH), ambos con una eficacia semejante ^(14)^. Sin embargo, el uso de estos medicamentos en niños puede provocar efectos adversos, como trastornos del sueño, disminución del apetito, dolor de cabeza, irritabilidad y dolor abdominal^(15)^.

Además, las barreras económicas dentro del sistema de salud dificultan la adherencia al tratamiento farmacológico. En Brasil, por ejemplo, la ausencia de estos medicamentos en la Lista Nacional de Medicamentos Esenciales (RENAME) impide su distribución gratuita, por lo que limita el acceso de los pacientes al tratamiento^(16-17)^.

Ante este escenario, familiares y profesionales de la salud han buscado enfoques alternativos que complementen la atención brindada a los niños con TDAH^(18-19)^. En este contexto, las terapias complementarias —definidas por el *National Center for Complementary and Integrative Health* (NCCIH), de los Estados Unidos, como enfoques no convencionales utilizados junto con la medicina tradicional para potenciar el tratamiento, se plantean como estrategias auxiliares en el manejo de los síntomas del TDAH^(20-21)^ .

Estas terapias incluyen prácticas como la acupuntura, la meditación, la fitoterapia y el yoga, entre otras, y están previstas en la Política Nacional de Prácticas Integradoras y Complementarias (PNPIC), implementada en el ámbito del Sistema Único de Salud (SUS) en 2006. Con base en esta política, el uso de terapias complementarias en Brasil creció un 70% en 2024, en comparación con 2022, sobre todo en la atención primaria y en servicios especializados mediante derivación profesional^(22-23)^.

Aunque estos enfoques aún enfrentan desafíos relacionados con la aceptación, prescripción e implementación en los servicios de salud, los estudios han demostrado beneficios significativos, especialmente en el tratamiento de niños con TDAH. Los principales resultados incluyen la reducción de los movimientos excesivos^(24)^, mejora de la atención selectiva y sostenida^(25)^, aumento de la concentración, desarrollo de habilidades motoras^(26)^ y mejora de la memoria^(27-28)^.

En cuanto a los profesionales de la salud, obstáculos como la falta de formación específica en estas prácticas, la ausencia de apoyo de los equipos, la inexistencia de protocolos clínicos estandarizados y la resistencia institucional a los abordajes no convencionales dificultan la aplicación efectiva de terapias complementarias en el manejo de niños con TDAH, incluso cuando los usuarios están interesados o existen evidencias favorables^(29-31)^.

Por ello, es fundamental conocer qué terapias complementarias han sido promovidas por los equipos de salud como parte de la atención integral a los niños con TDAH. Ante el creciente interés por los enfoques no farmacológicos y la necesidad de ampliar el conocimiento profesional sobre estas prácticas, la integración de la atención multidimensional en los servicios de salud es cada vez más relevante. Sin embargo, la evidencia sobre el tema aún es limitada, lo que resalta la importancia de nuevos estudios. En ese marco, este trabajo tuvo como objetivo identificar, en la literatura científica, terapias complementarias promovidas por profesionales de la salud para niños con TDAH.

## Método

### Tipo de estudio

Esta es una revisión de alcance realizada con base en las directrices metodológicas propuestas por el *Joanna Briggs Institute* (JBI)^(32)^ y por *Extension for Scoping Reviews* (PRISMA-ScR)^(33)^. El protocolo del estudio se registró en la plataforma *Open Science Framework* (OSF), disponible en osf.io/twdrh.

### Ubicación

La búsqueda bibliográfica se realizó en las siguientes bases de datos: *Medical Literature Analysis and Retrieval System Online* (MEDLINE/PubMed), *Excerpta Medica Database* (EMBASE), *Scopus*, *Web of Science* (WoS) y *Literatura Latino-Americana e do Caribe em Ciências da Saúde* (LILACS). Además, se realizó una búsqueda secundaria en *Google Scholar* y una búsqueda manual inversa sobre las referencias de los estudios primarios incluidos, con el objetivo de ampliar el mapeo.

### Periodo de estudio

La búsqueda se llevó a cabo entre mayo y junio de 2023. Se realizó una actualización de la búsqueda en abril de 2025 para garantizar que la revisión estuviese actualizada.

### Población

Se utilizó el mnemónico PCC, que representa población (P), concepto (C) y contexto (C), para definir el título y estructurar la pregunta de revisión, tal como recomienda el JBI para las revisiones de alcance^(32)^. En este estudio, la población estuvo constituida por niños de entre uno y nueve años, según la clasificación de edad de la Organización Mundial de la Salud^(34)^; el concepto engloba terapias complementarias realizadas por profesionales de la salud; y el contexto se centró en el diagnóstico del TDAH. Desde esta perspectiva, la pregunta guía se formuló de la siguiente manera: ¿Qué terapias complementarias promueve el equipo de salud para controlar el TDAH en los niños?

### Criterios de selección

Se incluyeron estudios que respondieron la pregunta guía; implicando a niños de entre uno y nueve años, aunque también incluyeran otros grupos de edad; publicados en portugués, inglés o español; sin restricciones en cuanto al año de publicación, diseño metodológico o tipo de material bibliográfico. También se consideraron estudios que abordaron el rango de edad establecido junto con otras edades.

Los criterios de exclusión fueron: resúmenes publicados en actas de eventos científicos; participantes con sospecha o diagnóstico de trastornos psiquiátricos comórbidos, como ansiedad, depresión, trastorno negativista desafiante, trastorno del espectro autista, entre otros; estudios orientados a la comparación entre la terapia complementaria y el tratamiento farmacológico; evaluación de la viabilidad del plan de contratación; y aquellos centrados exclusivamente en los mecanismos técnicos de la terapia. Estos criterios están numerados del 1 al 10 en el diagrama de flujo PRISMA (Figura 1).

### Definición de muestra

Las estrategias de búsqueda incluyeron descriptores con truncamientos apropiados, adaptados a cada base de datos, relacionados con la población, terapias complementarias y diagnóstico de TDAH. Entre los descriptores utilizados destacan: “child”, “children”, “complementary therapies”, “alternative medicine”, “alternative therapies”, “complementary medicine”, “attention deficit disorder with hyperactivity”, “ADHD”, “attention deficit hyperactivity disorder”, entre otros términos alternativos. La estrategia completa está disponible en el Apéndice Suplementario 1 (en https://doi.org/10.48331/scielodata.8ZOILU).

### Recopilación de datos

Los resultados de la búsqueda se ingresaron en el *software* Rayyan y luego se eliminaron los duplicados. La selección de títulos y resúmenes fue realizada por dos revisores de forma independiente, seguida de la lectura de los textos completos. Las divergencias fueron resueltas por un tercer revisor. Al final de este proceso se incluyeron 108 estudios en la revisión. Con la actualización realizada en abril de 2025, se agregaron 25 estudios más, con lo que se obtuvo un total de 133 publicaciones incluidas en el análisis.

### Variables de estudio

Las variables analizadas fueron: autor(es), año de publicación, país, idioma, objetivo(s), tipo de estudio, población, terapia complementaria, duración de la intervención y resultados principales.

### Procesamiento y análisis de datos

Para describir las características cuantitativas de los estudios se utilizó estadística descriptiva (frecuencias y porcentajes). Los resultados primarios se agruparon y presentaron en tablas, gráficos y figuras, con posterior discusión a la luz de las referencias identificadas.

### Aspectos éticos

Este estudio no requirió la aprobación de un Comité de Ética de Investigación.

## Resultados

La búsqueda en la base de datos identificó 1.444 estudios, de los cuales 415 eran duplicados y 72 se hallaban en idiomas no elegibles, por lo que el total se redujo a 957 artículos. De estos, 786 estudios fueron excluidos porque no cumplir los criterios de inclusión. Los 171 artículos restantes fueron leídos en su totalidad, lo que resultó en la exclusión de otros 85, dejando una muestra final de 86 estudios para la revisión.

Además, las búsquedas de la literatura gris mediante *Google Scholar* y el seguimiento de citas de los estudios incluidos identificaron 414 documentos, de los cuales 47 se incorporaron a la muestra final. Así, esta revisión incluyó un total de 133 estudios, como se ilustra en el diagrama de flujo PRISMA (Figura 1).

**Figura 1-:** Diagrama de flujo PRISMA para la selección de estudios. Fortaleza, CE, Brasil, 2025

En cuanto a las características de los estudios, como se muestra en la Tabla 1, los años de publicación oscilaron entre 1979 y 2024, siendo el 72,2% de los estudios publicados entre 2010 y 2024. Se observó un mayor número de publicaciones en los años 2024 (n=16), 2014 (n=14) y 2022 (n=10). Los estudios se concentraron principalmente en Asia (37,6%), América del Norte (30,9%) y Europa (26,3%). La estrategia de búsqueda utilizada en esta revisión no identificó publicaciones de Centroamérica. La revisión incluyó un total de 8.798 niños diagnosticados con TDAH, y el 51,1% de los estudios (n=68) tenían entre 10 y 100 participantes. El idioma predominante de las publicaciones fue el inglés (97,8%). En cuanto al diseño metodológico, 45 publicaciones fueron ensayos clínicos aleatorizados, 39 fueron revisiones (incluidos 11 metaanálisis), 11 fueron estudios experimentales y 6 fueron revisiones sistemáticas. Cabe señalar que los estudios clasificados como experimentales no detallaron el diseño metodológico utilizado. En el 46,6% de los artículos, el tiempo de intervención reportado osciló entre uno y seis meses.

**Tabla 1-:** Características de los estudios incluidos (n = 133). Fortaleza, CE, Brasil, 2025

**Características**	**Número (%)**
Año	
< 2000	7 (5,2%)
2000-2009	30 (22,6%)
2010-2024	96 (72,2%)
Región	
Asia	50 (37,6%)
América del Norte	41 (30,9%)
Europa	35 (26,3%)
Oceanía	2 (1,5%)
América del Sur	2 (1,5%)
África	3 (2,2%)
Idioma	
Inglés	130 (97,8%)
Español	2 (1,5%)
Portugués	1 (0,7%)
Diseño del estudio	
Ensayo clínico aleatorizado	45 (33,8%)
Revisión	39 (29,3%)
Estudio experimental	11 (8,3%)
Estudio piloto	6 (4,5%)
Estudio cualitativo	4 (3%)
Estudio de casos y controles	3 (2,3%)
Otros	25 (18,8%)
Número de participantes	
< 10	8 (6%)
10-100	68 (51,1%)
100-500	18 (13,6%)
> 500	4 (3%)
No informado	35 (26,3%)
Duración de las terapias complementarias	
≤ 1 mes	17 (12,8%)
1 a 6 meses	62 (46,6%)
≥ 6 meses	12 (9%)
No especificado	42 (31,6%)

Se identificaron sesenta y cinco terapias complementarias, agrupadas en cuatro categorías: terapias mente-cuerpo (n=158), intervenciones complementarias (n=60), fitoterapia (n=24) y terapia dietética (n=24), como se ilustra en la Figura 2. Entre las terapias mente-cuerpo, la más reportada fue *el neurofeedback* (n=38), seguido de la actividad física (n=20) y el yoga (n=13). La intervención complementaria más mencionada fue la suplementación con ácidos grasos poliinsaturados (n=14), seguida de zinc (n=10) y hierro (n=9). Otros suplementos incluyeron aminoácidos, gliconutrientes, ácido gamma-aminobutírico, glicina, L-teanina, L-tirosina, taurina, 5-hidroxitriptófano, S-adenosil -L-metionina (SAMe), fosfatidilserina, fosfatidilcolina y probióticos. Entre las terapias herbales o fitoterapia, se destacaron *Ginkgo biloba* (n=6), *Hypericum perforatum* (n=5) y Pycnogenol (n=4). Respecto a las terapias dietéticas se destacaron la dieta oligoantigénica (n=5) y la dieta restringida en azúcar (n=4).

Una tabla con características adicionales y las referencias de los estudios incluidos está disponible en el Material Suplementario 2 (en https://doi.org/10.48331/scielodata.WMW6W7).

**Figura 2-:** Terapias complementarias identificadas en los estudios y número de artículos por tipo de terapia. Fortaleza, CE, Brasil, 2025

## Discusión

Esta revisión de alcance identificó 65 modalidades de terapias complementarias empleadas por profesionales de la salud en el tratamiento de niños diagnosticados con TDAH, categorizadas en cuatro enfoques: terapias mente-cuerpo, intervenciones complementarias, medicina herbal o fitoterapia y terapia dietética.

Entre las terapias mente-cuerpo se destacaron *el biofeedback*, *el neurofeedback*, la actividad física y el yoga. El *biofeedback* es una técnica terapéutica que regula la respuesta del sistema nervioso autónomo, y promueve el equilibrio entre las funciones simpáticas y parasimpáticas. Esta terapia busca que el paciente alcance el autocontrol psicofisiológico^(35)^.

El *neurofeedback* es una subcategoría del *biofeedback* que actúa sobre el sistema nervioso central, entrenando al paciente para autorregular sus ondas cerebrales a través de la estimulación y observación de la actividad cerebral^(36-37)^. Los hallazgos sugieren una aparente eficacia del *biofeedback* en niños con TDAH, lo que resulta en una mejor atención^(38-40)^.

Sin embargo, un metaanálisis reciente no confirmó la eficacia del *neurofeedback* como tratamiento para el TDAH^(41)^. En una nueva revisión, se informó que los metanálisis concluyeron que el *neurofeedback* presenta una eficacia a corto plazo para los síntomas del TDAH^(42)^.

La actividad física es una práctica que aporta múltiples beneficios a la salud física y mental^(43)^. El ejercicio aeróbico ha sido evaluado como una intervención terapéutica capaz de aliviar los síntomas del TDAH y promover mejoras en la memoria y las funciones ejecutivas^(27,44)^. El ejercicio aeróbico, como bailar, nadar, caminar y correr, parece reducir la falta de atención, la impulsividad y la hiperactividad en niños con TDAH^(28)^.

Un metaanálisis reciente reveló que la actividad física mejora la concentración y promueve el desarrollo de habilidades motoras^(45)^. En particular, la actividad física tiene un efecto más expresivo en comparación con otras modalidades de tratamiento no farmacológico^(26)^. Los hallazgos refuerzan la actividad física como una estrategia complementaria eficaz para controlar los síntomas del TDAH en poblaciones pediátricas.

Otra terapia ampliamente discutida en la literatura es el yoga, basada en una serie de ejercicios orientados al equilibrio físico y mental, la cual consiste en un conjunto de técnicas y conocimientos orientados al desarrollo personal, crecimiento psicológico y bienestar espiritual^(46)^. Un estudio experimental con evaluación pre y post intervención sobre la práctica de yoga en niños con TDAH informó efectos positivos, incluida la reducción de movimientos como balancear las manos y/o los pies y la mejora de las conductas interruptivas e intrusivas durante otras actividades^(47)^.

Se observaron mejoras notables en la atención selectiva y sostenida, así como en las habilidades de discriminación, después de la práctica de yoga en niños con TDAH^(24)^. Así, estudios recientes indican una actitud positiva hacia el uso del yoga como terapia complementaria para el TDAH.

Otras prácticas complementarias como *el biofeedback* , la meditación, la homeopatía, la quiropráctica, la acupuntura, la masoterapia, el *Tai Chi Chuan*, la hipnoterapia, la terapia de juego, el *mindfulness*, el entrenamiento de relajación conductual, el entrenamiento de la función cognitiva, la electroacupuntura, la estimulación magnética transcraneal repetitiva, el ruido blanco, la terapia antroposófica, la arteterapia, la kinesiología aplicada, Cogmed, el *Qigong*, la euritmia terapéutica, las flores de Bach, el método Tomatis de entrenamiento sonoro, la musicoterapia, la terapia manipulativa osteopática, la hipoterapia, la dieta sensorial y la estimulación eléctrica transcutánea de puntos de acupuntura presentaron evidencia no concluyente en la población pediátrica. Esto pone de relieve la necesidad de realizar más investigaciones para demostrar su eficacia.

Entre las intervenciones suplementarias identificadas, se destaca el uso de ácidos grasos poliinsaturados, compuestos por cadenas largas con múltiples enlaces dobles, siendo los principales representantes los omega-3 y omega-6. Las principales fuentes de estos suplementos incluyen salmón, aceite de onagra y aceites vegetales como el aceite de maíz^(25,48-49)^.

Un ensayo clínico aleatorizado, doble ciego y controlado con placebo realizado en Canadá demostró que los ácidos grasos omega-3 mostraron una tendencia estadísticamente significativa en la reducción de los síntomas centrales del TDAH en niños diagnosticados con el trastorno^(50)^. Del mismo modo, otro estudio observó mejoras graduales en el rendimiento académico de los niños^(51)^. Sin embargo, un ensayo clínico controlado con placebo en niños italianos diagnosticados con TDAH no encontró diferencias significativas en el aprendizaje o la reducción de la falta de atención entre los grupos que recibieron suplementos de omega-3 y omega-6 en comparación con placebo^(52)^. En ese contexto, se advierte que no hay evidencia científica suficiente para respaldar el uso de omega-3 y omega-6 como terapia aislada para el TDAH.

Otros componentes descritos en la literatura incluyen zinc y hierro, minerales directamente asociados con el desarrollo físico y neurológico. Estos minerales cumplen varias funciones, siendo el zinc esencial para procesos celulares como la síntesis de proteínas y ADN, y el hierro fundamental para la producción de hormonas como la dopamina y la noradrenalina^(53)^.

Se ha demostrado que la suplementación con zinc es eficaz para reducir la hiperactividad y la impulsividad en los niños^(54-56)^. No obstante, su eficacia parece estar relacionada con la dosis, lo que requiere más investigación científica^(57-58)^. Un ensayo clínico aleatorizado con 60 niños asiáticos mostró que la suplementación con zinc no produjo diferencias significativas entre los grupos antes y después de la intervención, lo que sugiere una falta de eficacia para este tipo de terapia complementaria^(59)^.

Respecto a la suplementación con hierro, un ensayo clínico aleatorizado observó que los niños que recibieron esta intervención mostraron avances en el tratamiento del TDAH, especialmente en las subescalas de hiperactividad, impulsividad e inatención^(60)^. Sin embargo, otra revisión indicó que no hay suficiente evidencia científica para recomendar la suplementación con hierro en niños con TDAH que no presentan deficiencia de este mineral^(61)^. A pesar de la diversidad de literatura sobre suplementación, aún falta evidencia sobre la efectividad de estos minerales como terapias con impacto positivo en el tratamiento del TDAH.

Las intervenciones complementarias han mostrado resultados mixtos con el uso de magnesio, acetil L-carnitina, vitamina B6, melatonina, vitamina D, dimetilaminoetanol, calcio y magnesio, yodo, gliconutrientes, ácido gamma- aminobutírico, glicina, L-teanina , L-tirosina, taurina, 5-hidroxitriptófano, S-adenosil-L-metionina (SAMe), fosfatidilserina y fosfatidilcolina. Aunque algunos estudios han reportado beneficios en la reducción de los síntomas del TDAH, la evidencia aún es insuficiente para su uso rutinario en la práctica clínica y se necesitan más estudios controlados. Es de destacar que no se encontró respaldo científico que justifique el uso de gliconutrientes en el tratamiento del TDAH.

El *Ginkgo biloba* es un medicamento a base de hierbas con propiedades antioxidantes y antiinflamatorias, similar a la clase farmacológica de los nootrópicos, sustancias que actúan sobre el sistema nervioso central para mejorar las funciones cognitivas^(62)^. Se utiliza en el tratamiento de diversas disfunciones cognitivas, como demencia, insuficiencia cerebrovascular, pérdida de memoria reciente, dolor de cabeza, mareos y tinnitus^(63)^.

El *Ginkgo biloba* mejora la respuesta al tratamiento clínico, aunque sus efectos se limitaron a los síntomas de falta de atención en un estudio de seis semanas^(64)^. Sin embargo, a pesar de los efectos prometedores sobre los síntomas del TDAH, los resultados no fueron concluyentes en otro estudio^(65)^.

En el área de la fitoterapia se identificaron plantas como *Hypericum perforatum*, *Pinus pinaster*, *Rhodiola rosa*, *Matricaria recutita L.*, *Valeriana officinalis* y *Bacopa monnieri.* Actualmente, no hay suficiente evidencia científica para recomendar estas plantas como terapias complementarias efectivas para el TDAH, y se necesita más investigación.

La dieta oligoantigénica se funda en el consumo de alimentos sin aditivos químicos, como colorantes y conservantes, que pueden desencadenar los síntomas del TDAH al actuar como potenciales antígenos o alérgenos alimentarios. Los alimentos frecuentemente asociados con reacciones alérgicas incluyen la leche de vaca, el queso, los huevos, el chocolate y las nueces^(66)^.

La evidencia sobre la restricción de colorantes alimentarios artificiales ha avanzado hasta el punto en que se considera una práctica basada en la evidencia^(67)^. Los resultados sugieren que esta dieta, aplicada durante 4 semanas, generó mejoras duraderas en los síntomas del TDAH, siendo considerada una terapia complementaria válida al ser reevaluada aproximadamente 3 años después de la intervención^(68)^. Esta terapia puede servir como recurso auxiliar para mejorar los síntomas en niños con TDAH.

Se mencionaron otras cuatro prácticas dietéticas: la dieta restringida en azúcar, la dieta Feingold, la dieta megavitamínica y la dieta cetogénica. Los estudios han arrojado resultados contradictorios y no concluyentes para las dietas Feingold y cetogénica. Cabe destacar que los estudios no han recomendado la terapia con megavitaminas debido a los posibles efectos adversos asociados con este enfoque dietético.

El TDAH plantea desafíos importantes relacionados con cambios motores, perceptivos, cognitivos y conductuales. Por lo tanto, se deben adoptar enfoques específicos según la edad para garantizar un manejo y cuidado adecuados^(69)^.

En la primera infancia, especialmente alrededor de los 12 meses de edad, se hacen evidentes cambios en la actividad motora, la expresividad emocional y el desarrollo del lenguaje^(70)^. Entre los 3 y los 7 años, aparecen los primeros signos indicativos de TDAH, aunque el diagnóstico formal se produce más tarde. En esta etapa es común que se manifiesten conductas hiperactivas. Las estrategias recomendadas para esta etapa incluyen establecer rutinas consistentes y adoptar métodos de enseñanza más estructurados^(11)^.

El TDAH generalmente se diagnostica alrededor de los 7 años, debido a dificultades académicas o de comportamiento. Estudios comparativos han demostrado que los niños con TDAH tienen un peor desempeño que sus pares en los dominios social, emocional y académico^(11)^.

Durante el desarrollo de este estudio se identificaron varias limitaciones, como restricciones lingüísticas en la selección de fuentes, ausencia de datos relevantes en algunos estudios y artículos ilegibles. Tales limitaciones impidieron un análisis más profundo de la evidencia.

El papel de enfermería en las unidades de atención primaria de salud puede influir positivamente en la efectividad de las terapias complementarias a través de la educación para la salud dirigida a padres e hijos con TDAH. Este proceso educativo, combinado con consultas que fomentan la participación activa de la familia en la definición de expectativas, contribuye a una mayor adherencia al tratamiento y al fortalecimiento del vínculo terapéutico con el paciente^(71)^.

Además, este papel se extiende al ámbito escolar, donde los profesionales de enfermería realizan el acompañamiento del niño, considerando aspectos comportamentales, psicosociales y pedagógicos^(72-73)^.

Se espera que los enfermeros, junto con otros profesionales de la salud, puedan incorporar estas prácticas en sus consultas como un recurso complementario al tratamiento farmacológico. Estos enfoques se pueden adaptar a las necesidades individuales de cada paciente, con el objetivo de brindar una atención integral y mejorar la calidad de vida a lo largo del tratamiento.

## Conclusión

El uso de terapias complementarias muestra un potencial prometedor para mejorar los síntomas del TDAH infantil, con especial énfasis en las terapias mente-cuerpo, como *neurofeedback*; en el campo de la suplementación, el uso de ácidos grasos poliinsaturados, zinc y hierro; en fitoterapia, el uso de *Ginkgo biloba*; y, entre las terapias dietéticas, la dieta oligoantigénica.

Estas intervenciones pueden contribuir a mejorar la atención, la regulación del comportamiento, las habilidades motoras y el rendimiento académico en niños diagnosticados con TDAH, abordando aspectos relacionados con la hiperactividad, la impulsividad y la inatención.

Sin embargo, debido al número limitado de estudios y la falta de investigaciones recientes sobre ciertas terapias, la evidencia sobre la efectividad de algunas de estas prácticas aún no es concluyente.

El desarrollo de estudios experimentales, especialmente centrados en las terapias menos utilizadas, puede proporcionar una base teórica para el uso seguro y la recomendación de estas prácticas en el ejercicio profesional. Además, estos estudios pueden contribuir a obtener mejores resultados en el tratamiento del TDAH infantil.

## Data Availability

El conjunto de datos de este artículo está disponible en la página de la RLAE en el repositorio SciELO Data, en los enlaces https://doi.org/10.48331/SCIELODATA.8ZOILU y https://doi.org/10.48331/SCIELODATA.WMW6W7.
